# Categorical discrimination of human body parts by magnetoencephalography

**DOI:** 10.3389/fnhum.2015.00609

**Published:** 2015-11-04

**Authors:** Misaki Nakamura, Takufumi Yanagisawa, Yumiko Okamura, Ryohei Fukuma, Masayuki Hirata, Toshihiko Araki, Yukiyasu Kamitani, Shiro Yorifuji

**Affiliations:** ^1^Department of Functional Diagnostic Science, Osaka University Graduate School of MedicineSuita, Japan; ^2^Department of Neurosurgery, Osaka University Graduate School of MedicineSuita, Japan; ^3^Department of Neuroinformatics, ATR Computational Neuroscience LaboratoriesKyoto, Japan; ^4^Japan Science and Technology Agency, Precursory Research for Embryonic Science and TechnologyOsaka, Japan; ^5^Graduate School of Information Science, Nara Institute of Science and TechnologyIkoma, Japan; ^6^Graduate School of Informatics, Kyoto UniversityKyoto, Japan

**Keywords:** visual cortex, body perception, decoding, categorization, magnetoencephalography

## Abstract

Humans recognize body parts in categories. Previous studies have shown that responses in the fusiform body area (FBA) and extrastriate body area (EBA) are evoked by the perception of the human body, when presented either as whole or as isolated parts. These responses occur approximately 190 ms after body images are visualized. The extent to which body-sensitive responses show specificity for different body part categories remains to be largely clarified. We used a decoding method to quantify neural responses associated with the perception of different categories of body parts. Nine subjects underwent measurements of their brain activities by magnetoencephalography (MEG) while viewing 14 images of feet, hands, mouths, and objects. We decoded categories of the presented images from the MEG signals using a support vector machine (SVM) and calculated their accuracy by 10-fold cross-validation. For each subject, a response that appeared to be a body-sensitive response was observed and the MEG signals corresponding to the three types of body categories were classified based on the signals in the occipitotemporal cortex. The accuracy in decoding body-part categories (with a peak at approximately 48%) was above chance (33.3%) and significantly higher than that for random categories. According to the time course and location, the responses are suggested to be body-sensitive and to include information regarding the body-part category. Finally, this non-invasive method can decode category information of a visual object with high temporal and spatial resolution and this result may have a significant impact in the field of brain–machine interface research.

## Introduction

Neural decoding of visual recognition has been developed to understand how the information of an image is coded in the human brain. Many studies have demonstrated that the contents of visual recognition can be inferred from brain signals obtained either invasively or non-invasively. For example, the electrocorticography (ECoG) signals responded characteristically to visual stimuli of several categories such as the face and body (Allison et al., 1994; Engell and McCarthy, 2014). Using a decoding method, the visual object category was successfully classified with the ECoG signals (Majima et al., 2014). Moreover, even non-invasive signals, such as functional magnetic resonance imaging (fMRI) and magnetoencephalography (MEG), were successfully decoded to infer the presented images of arbitrary characters, the contents of dreaming (Kamitani and Tong, 2005; Miyawaki et al., 2008; Horikawa et al., 2013), and the visual object category (Martin et al., 1996; Gauthier et al., 2000; Carlson et al., 2003; Kiani et al., 2007; Kriegeskorte et al., 2008; DiCarlo et al., 2012; Van de Nieuwenhuijzen et al., 2013; Cichy et al., 2014). The decoding method reveals how visual information was encoded and how it is processed in the brain (Peelen and Downing, 2007).

Among the numerous types of visual object recognition, the discrimination of body parts, including the face, is important for humans and has been studied well previously (Allison et al., 2000; Downing et al., 2001; Rizzolatti et al., 2001; Buccino et al., 2004; Peelen and Downing, 2005, 2007; Thierry et al., 2006; Taylor et al., 2010). In macaques, single neurons in the inferior temporal (IT) cortex respond to specific categories of body parts representing the category information (Desimone et al., 1984; Tsao et al., 2006). In humans, visual representation of the face and other body parts selectively activates the fusiform body area (FBA), fusiform face area (FFA), and extrastriate body area (EBA) located at the posterior fusiform gyrus and at the posterior end of the IT sulcus, respectively, as observed by fMRI (Downing et al., 2001; Peelen and Downing, 2005; Bracci et al., 2010; Orlov et al., 2010). In transcranial magnetic stimulation (TMS) studies, the EBA was demonstrated as the essential region for the perception or discrimination of human body parts except the face (Urgesi et al., 2004, 2007; Pitcher et al., 2009). Moreover, it has been demonstrated that the FFA selectively responds to human faces (Kanwisher et al., 1997; McCarthy et al., 1997; Gauthier et al., 1999; Engell and McCarthy, 2014).

Using MEG and electroencephalography, the visual image of the face and body was shown to activate a selective response known as M170 and M190, respectively (Thierry et al., 2006; Peelen and Downing, 2007; Ishizu et al., 2010; Gao et al., 2013; Cichy et al., 2014). However, it remains unclear whether these body perception responses are selective to a particular category of body parts, such as the hand or the foot. Here we performed a decoding analysis using the MEG signals recorded while viewing body parts to reveal the temporal and spatial distribution of category information related to human body parts.

Magnetoencephalography signals were recorded while the subjects viewed several images of three categories of body parts (foot, hand, or mouth) or non-human objects. To elucidate responses characteristic to the body parts, we analyzed these signals through a source reconstruction method using Variational Bayesian Multimodal EncephaloGraphy (VBMEG; Sato et al., 2004; Yoshioka et al., 2008) and a decoding method using a support vector machine (SVM). For all subjects, we observed characteristic MEG responses with dipole patterns on the occipitotemporal cortex from 140 to 240 ms after the presentation of images. Using VBMEG, signal sources were identified mainly in the EBA and FFA. Notably, cortical activities in these areas varied significantly, responding differently to the three types of body parts. Moreover, the three categories of body parts were successfully classified using MEG signals around 190 ms after the visual stimulations. Therefore, the category of body part was non-invasively decoded using MEG signals corresponding to a body-sensitive response. Here we show that even a non-invasive method can decode the category information of the visual object with high temporal and spatial resolution.

## Materials and Methods

### Subjects

Nine healthy subjects (one male and eight females; mean age ± SD: 24.3 ± 5.0 years) participated in this study. All were right-handed (as assessed by the Edinburgh Handedness Inventory), had normal or corrected-to-normal vision, and had no history of neurological or psychiatric disorders. The experiments were conducted according to the principles of the Declaration of Helsinki, and the experimental procedures were approved by the Ethics Committee of Osaka University. Informed consent to participate in the study was obtained from all subjects.

### Visual Stimuli

**FIGURE 1:**
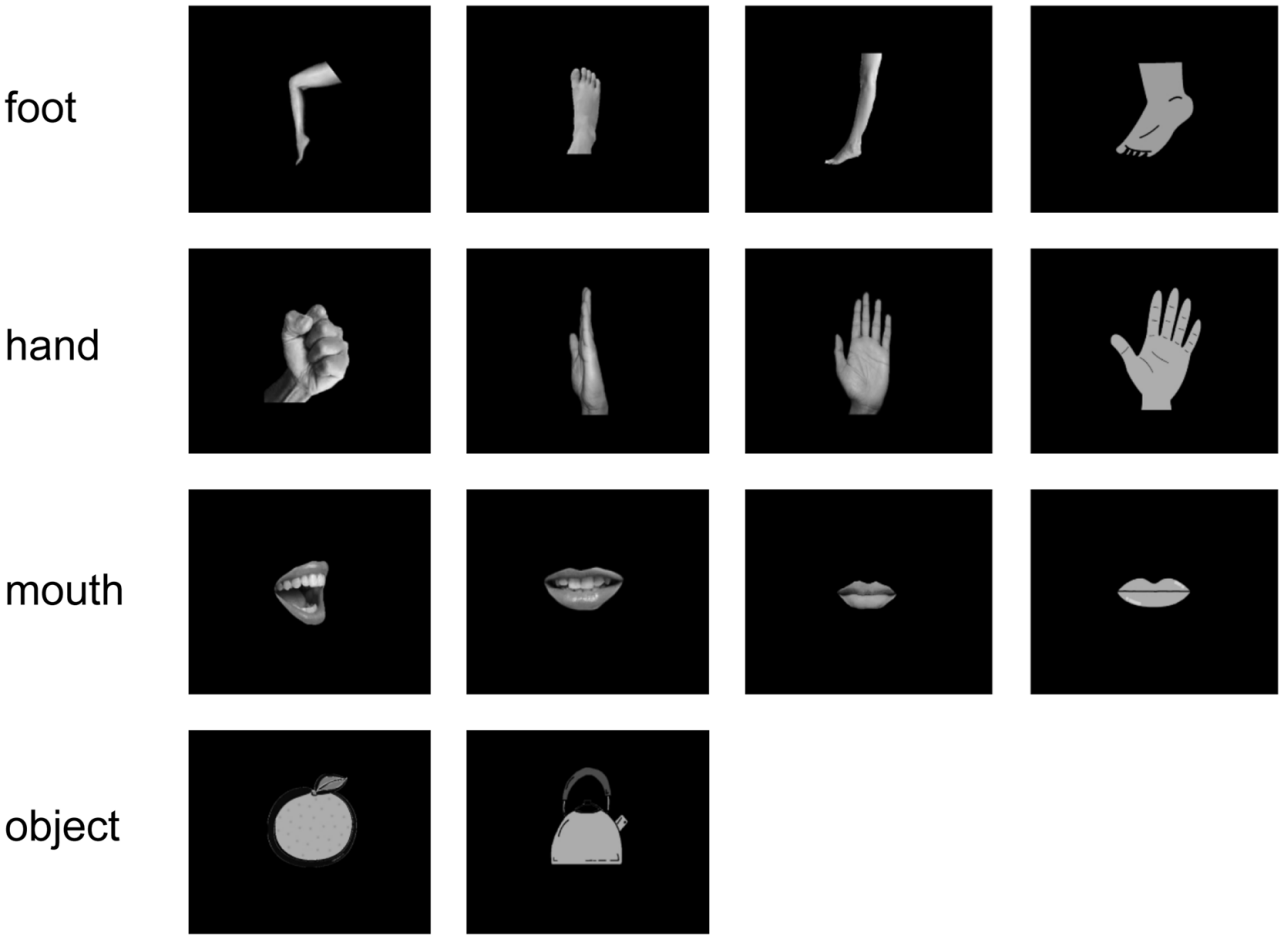
**Visual stimulus set.** We used 14 white-on-black images of body parts and other objects. There were four images of each body part and two images of objects.

All subjects were instructed to watch visual stimuli, while the MEG signals were recorded. The stimuli consisted of 14 simple white-on-black pictures showing four types of hands, four types of feet, four types of mouths, and two types of objects. The pictures of stimuli are shown in **Figure 1**. Each picture was presented 40 times (in total 560 presentations per subject). For each picture presentation, a fixation point was presented for 1000 ms before the picture itself was presented for 500 ms. Pictures were presented in a pseudo-random order.

Pictures were displayed on a projection screen 325 mm away from the subjects’ eyes using a visual presentation system (Presentation, Neurobehavioral Systems, Albany, CA, USA) and a liquid-crystal projector (LVP-HC6800, Mitsubishi Electric, Tokyo, Japan). The luminance of each image was adjusted to a value of 7 to prevent any bias due to luminosity. To reduce contamination from muscle activities and eye movements, we instructed the subjects to not move their shoulders and watch the center of the display without moving their eyes or blinking. Additionally, to monitor the attention paid by the subjects to stimuli, we instructed the subjects to press the buttons assigned to each body-part category when prompted by a directive. This happened 40 times per task. Some apparent artifacts were removed before the oﬄine analysis.

### MEG Recordings

Neuromagnetic activities were recorded in a magnetically shielded room with a 160-channel whole-head MEG system equipped with coaxial type gradiometers (MEG Vision NEO, Yokogawa Electric Corporation, Kanazawa, Japan). The subject was lying supine on a bed with his/her head centered. Before and after recording, the head position was measured with five coils placed on face (two at the external meatus of each ear and three points on the forehead). Data were sampled at a rate of 1000 Hz and filtered with an online low-pass filter at 200 Hz. After data acquisition, a notch filter at 60 Hz was applied to eliminate the AC line noise.

### Analysis of MEG Data

We analyzed epochs from -500 ms before stimulus onset to 500 ms after stimulus onset and applied a band pass filter from 1 to 30 Hz to the MEG signals. The beginning of the visual presentation of the picture is referred as time 0 ms. The baseline correction was made using the epoch between -500 and -100 ms before stimulus onset. We used 120 channels, except for the frontal cortex channel, to remove noise attributable to blinking.

The amplitudes and latencies in the recoded signals to stimulus were compared for each category to investigate whether body-sensitive responses were elicited. For the analyzed period, isomagnetic fields were obtained for each subject. Generally, isomagnetic fields show a dipole pattern centered around the occipitotemporal cortex. We call each location of single-current dipole as “vertex”. The sensor with the maximum negative component was chosen, and its component was defined as the peak amplitude in each subject; the timing of the peak amplitude was defined as the peak latency. The mean amplitude and latency of all subjects were compared among categories.

The average cortical currents of all subjects in each category were estimated using VBMEG from the selected signals. We reconstructed the cortical surface using FreeSurfer image analysis (Dale et al., 1999). With VBMEG, we estimated 4004 single-current dipoles that were equidistantly distributed on and perpendicular to the cortical surface. The method calculated an inverse filter to estimate the cortical current for each dipole from MEG sensor signals (Fukuma et al., 2015). The inverse filter was estimated from MEG signals during the time when the body-sensitive responses were observed. The filter was then applied to sensor signals in each trial to calculate cortical currents. The estimated cortical currents were time-averaged with 20-ms time window for each vertex.

The time-averaged estimated cortical currents of single trails of each participant at each location were compared by one-way analysis of variances (ANOVAs) among the three categories of different body parts (foot, hand, and mouth) or two categories from the three body parts (foot/hand, foot/mouth, and hand/mouth). At each of 4004 locations on the cortex, we obtained 160 values of time-averaged estimated cortical currents for each category of the three body parts. The 160 values of the three or two groups were compared by one-way ANOVA to obtain *F*-value. Notably, we performed ANOVA four times for each subject. One is the ANOVA of three categories of different body parts. The others are the ANOVA of two categories for each; (1) foot and hand, (2) foot and mouth, and (3) hand and mouth. The *F*-value of ANOVA was estimated for each location. Then, we averaged the *F*-values for each location among all subjects for each comparison. The averaged *F*-values were color-coded on the normalized brain surface.

Finally, we calculated the decoding accuracies from the MEG responses. MEG signals of each sensor were averaged in a 20-ms time window slid by 10 ms for the period from -100 to 500 ms. Then, these time-averaged amplitudes of each sensor were used as inputs for the decoding algorithms. The decoding accuracies of the estimates were evaluated using a 10-fold cross-validation (Yanagisawa et al., 2009). Using the obtained features, a linear classifier was estimated by the SVM to infer the category of the body part presented on a trial-by-trial basis (Fukuma et al., 2015). The classifier calculated the linearly weighted sum of the 20-ms time-averaged features of MEG signals plus bias for each class. Then the class with the maximum value was chosen as the inferred class. Individual weights and bias for each class were determined using the SVM applied to a training data set. The SVM algorithm was implemented using Matlab 2012a (Mathworks; Natic, MA, USA).

Here we performed two types of decoding for the same MEG responses. First, in the “categorical class”, the MEG responses for the 12 images of body parts were divided into three categorical classes of body parts. Each class consists of the responses for four types of images belonging to a single body-parts category (four images of each row in **Figure 1**). The body-part category was inferred by MEG responses using the decoding method. On the other hand, in the “random class,” the same MEG responses for twelve images of body parts were divided into three random classes. Each class consists of four types of image belonging to three categories of body parts (e.g., one image of foot, one of hand, and two of mouth). Each of the three groups has responses for the three body parts. The randomly assigned groups were classified by the MEG responses using the same decoding method. Notably, each of the decoding method classified the MEG responses into three groups (3-choice classification). Then, we compared the decoding accuracy between the categorical class, and the random class to evaluate whether the MEG responses have the information of body-part category.

## Results

**FIGURE 2:**
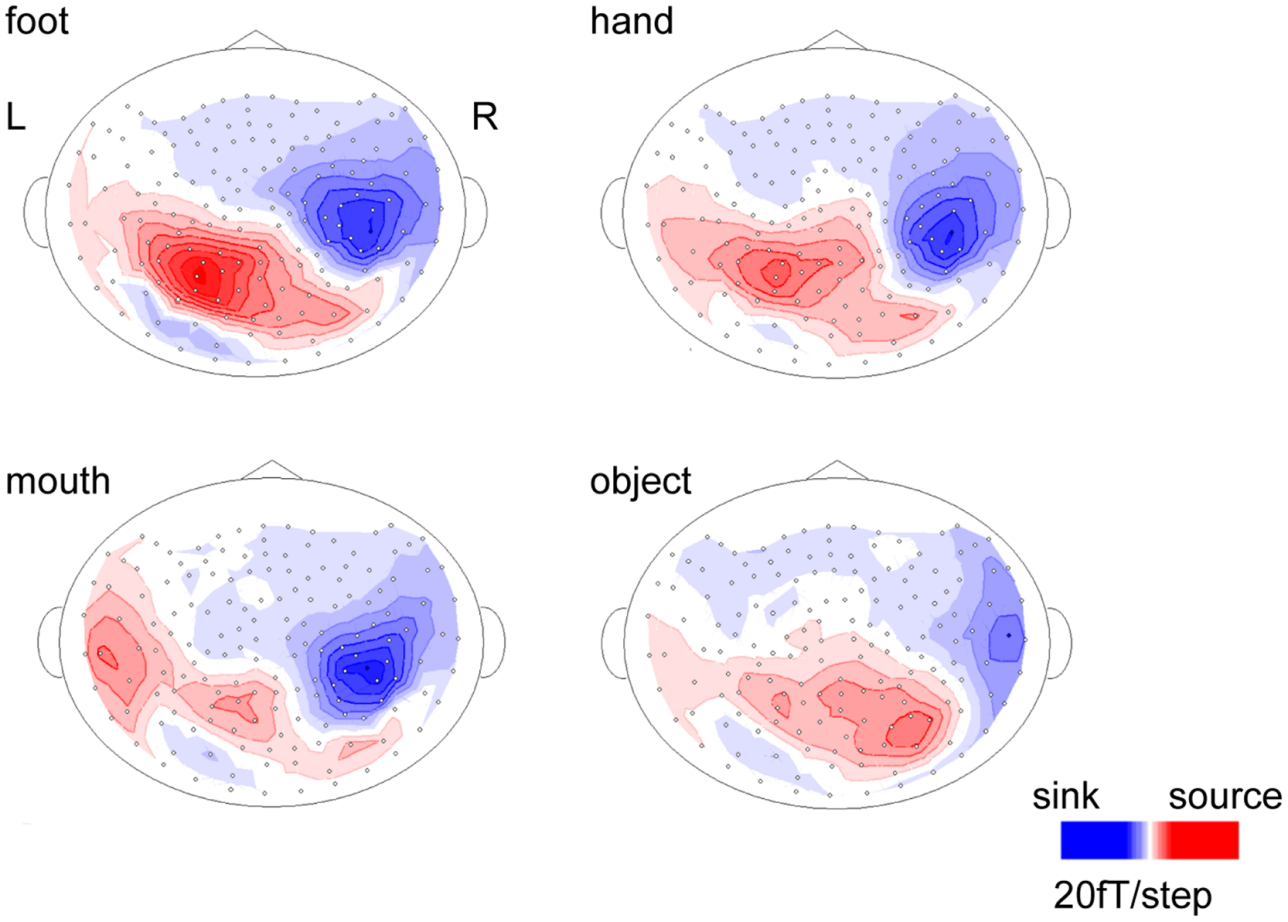
**Isomagnetic fields of a representative subject at the peak latencies of each category.** In all subjects, similar dipole patterns were observed between 160 and 240 ms.

**FIGURE 3:**
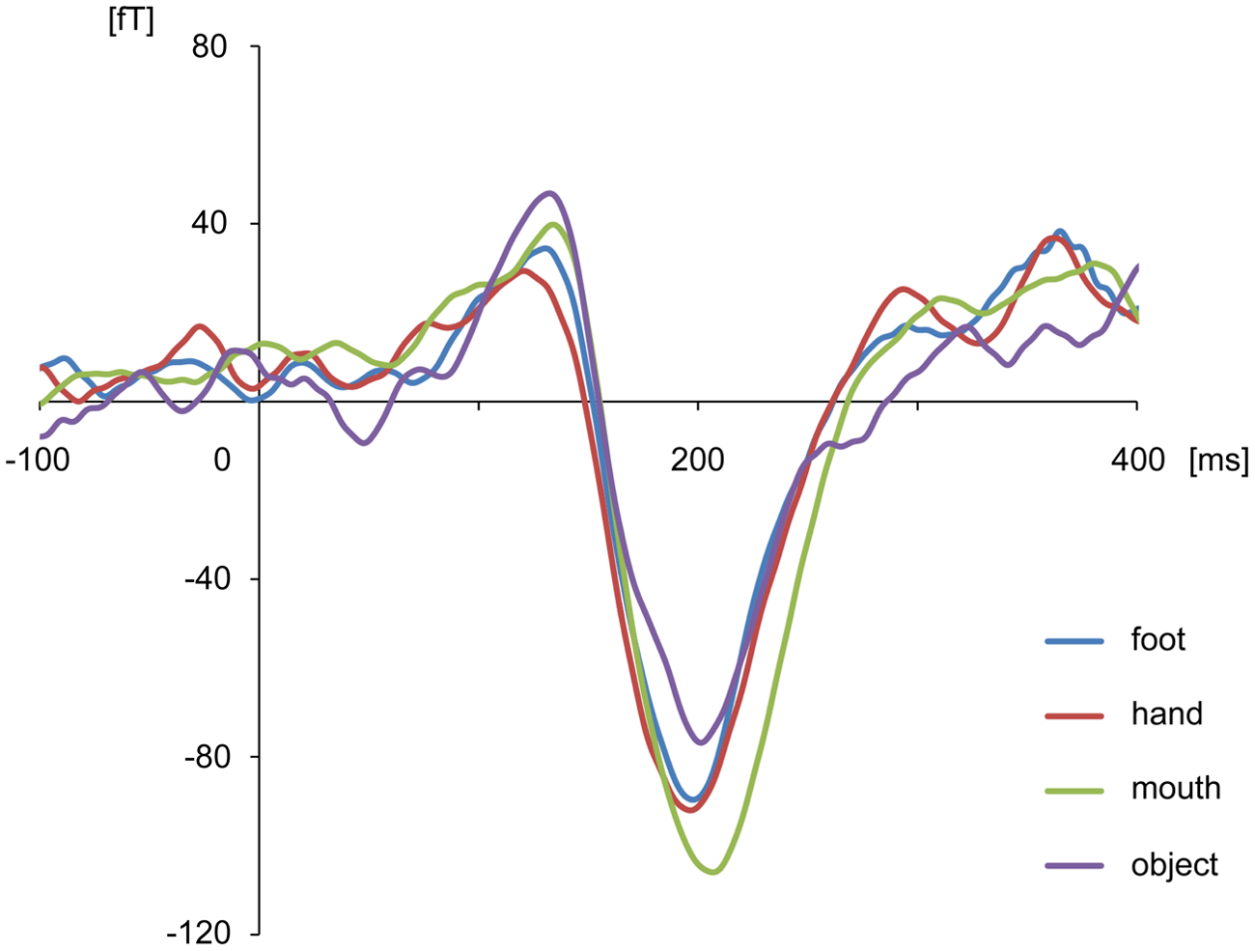
**Average waveform of the selected channels in each category for all subjects.** There were no differences among categories in peak amplitudes and latencies.

The body-sensitive responses were captured by MEG signals while the subjects were viewing four types of images representing three body parts. The isomagnetic fields from 140 to 240 ms after stimulus onset showed a clear dipole pattern centered on the occipitotemporal cortex for all subjects (**Figure 2**). The largest mean amplitude was evaluated for each category of visual images: mouth, hand, foot, or objects. The timing of the peak amplitudes (latency) and the peak amplitudes were not significantly different among the categories of visual images (one-way ANOVA; latency, *p* = 0.61, amplitude, *p* = 0.42; **Figure 3**).

**FIGURE 4:**
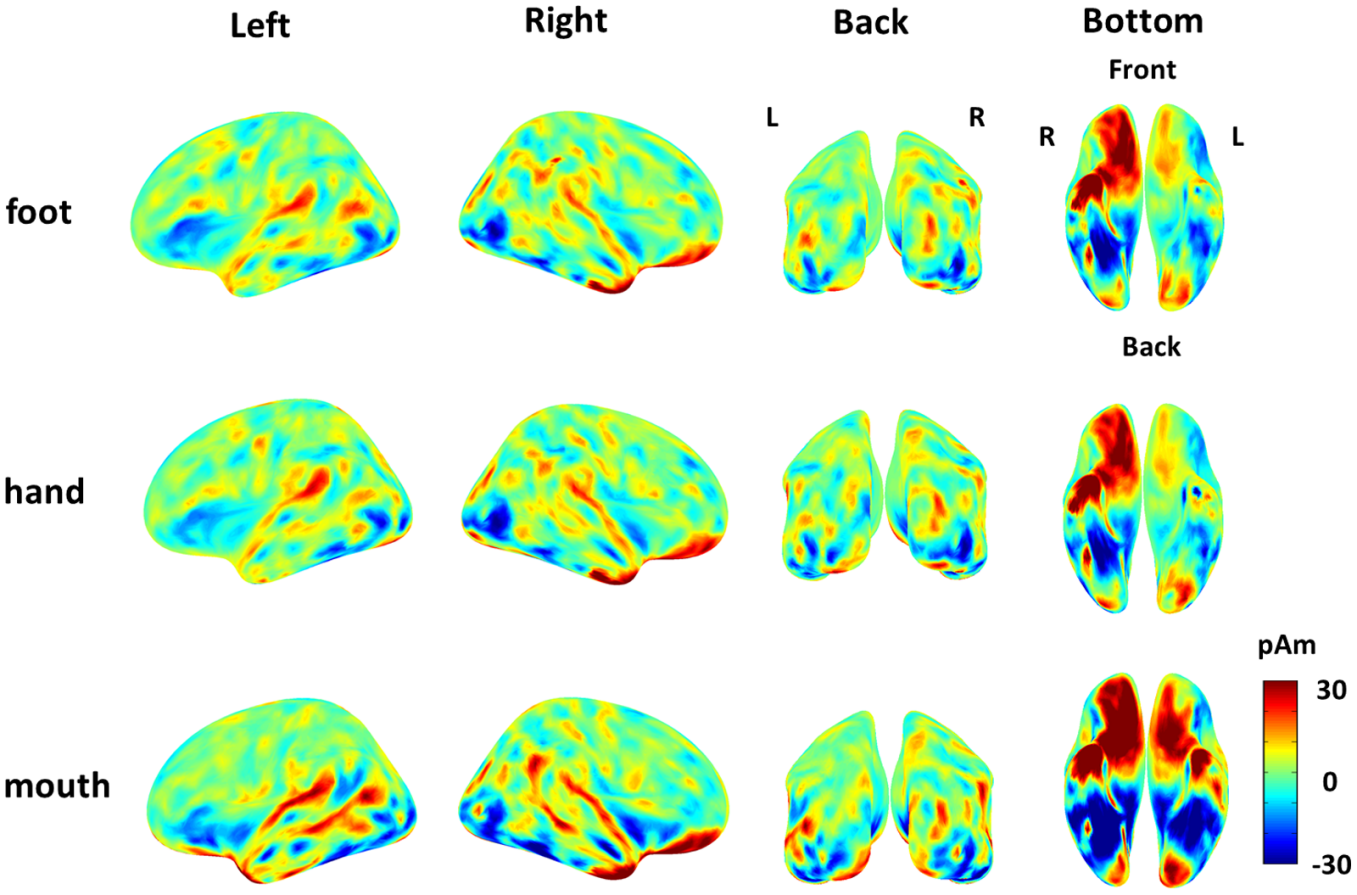
**Average cortical current of responses to each body part from 180 to 200 ms after stimulus onset.** The average cortical currents from 180 to 200 ms are color-coded on the normalized brain surface. The cortical currents estimated from MEG signals of each subject and averaged from 180 to 200 ms. The time-averaged cortical currents were averaged within the three different body-part categories for each subject. Then, those averaged cortical currents were averaged among all subjects. The activation were observed around the FBA and EBA in each category.

The cortical currents were estimated from the MEG signals by the source reconstruction technique using VBMEG to identify the cortical area sensitive to the category of body part. Those were color-coded on the normalized brain. The average cortical potential of all subjects was highest around the FBA and EBA at approximately 190 ms after stimulus presentation (**Figure 4**).

**FIGURE 5:**
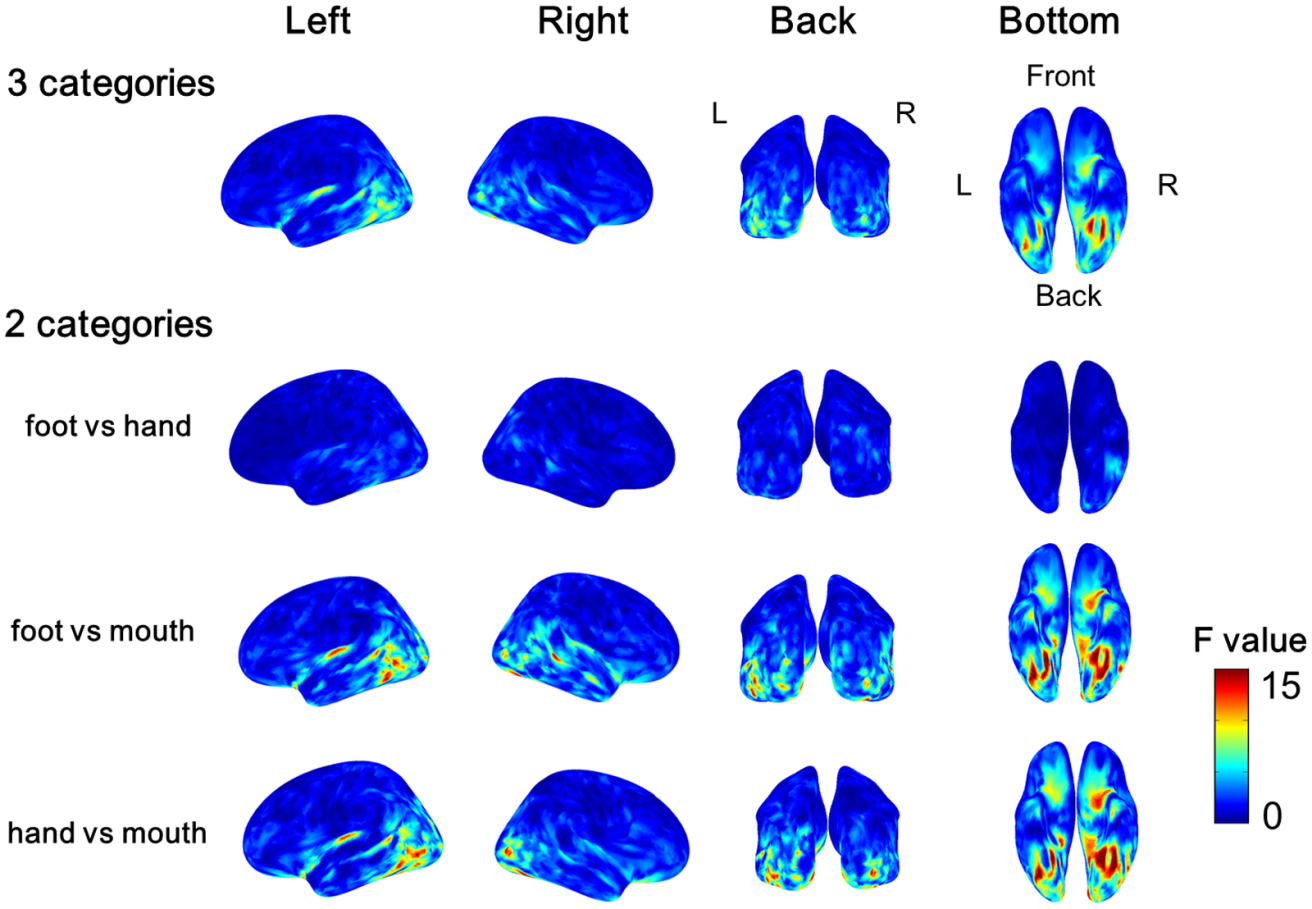
**Average *F*-values of all subjects calculated by one-way ANOVA among categories of body parts.** The *F*-value of one-way ANOVA was evaluated among three categories or two categories of the body parts at each vertex of cortical surface in each subject. Then we averaged all *F*-values in each subject. The group average *F*-values of the three Body part categories and of the contrast between pairs of two body parts are color-coded on normalized brain.

The variance of the cortical currents among the three categories or two categories of body parts was evaluated by one-way ANOVA. The *F*-values were averaged among all subjects and color-coded on the reconstructed surface of the normalized brain. Significant high *F*-values were obtained around the EBA and FBA (**Figure 5**). The high *F*-values on the occipitotemporal cortex clearly demonstrated that the cortical currents significantly varied between at least two types of body parts. Moreover, showing foot/hand, foot/mouth, and hand/mouth combinations induced similar pattern of results, although the variances were larger when the “mouth” category was included. These results suggest that the cortical currents on the occipitotemporal cortex are activated selectively for categories of body parts.

**FIGURE 6:**
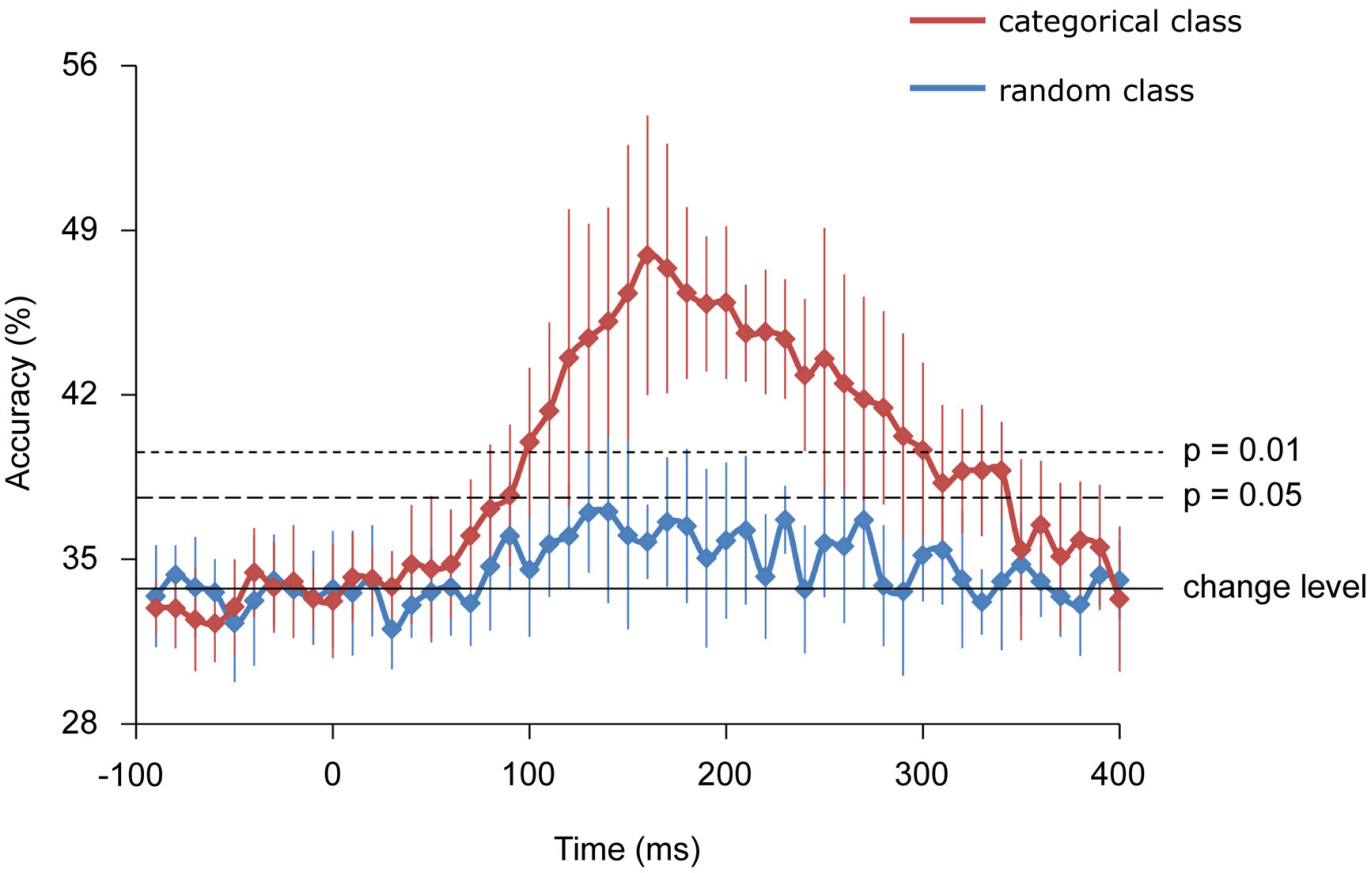
**Classification accuracies.** Error bars indicate 95% confidence intervals. The accuracy of the categorical class from 160 to 240 ms was significantly higher than that of random class (Student *t*-test; *p* < 0.01).

Further, using decoding techniques, body-part categories, including different types of visual images, were successfully classified by the MEG signals of each single trial. Starting from 100 and peaking at 160 ms, the accuracy of “categorical class” was 47.9 ± 5.9% for classifying three categories of body images and significantly exceeded the rate that would occur by chance (33.3%), (*n* = 9, binomial test; *p* < 0.01). Alternatively, the accuracy of “random class” peaking with 35.7 ± 1.6% did not significantly exceed chance level (*n* = 9, binomial test; *p* > 0.05). Moreover, the accuracy of “random class” was significantly lower compared with that of “categorical class” from 160 to 240 ms (*n* = 9, student’s *t*-test; *p* < 0.01; **Figure 6**).

## Discussion

This study has demonstrated that the MEG signals during perception of body images significantly varied among each category of the body part at a level suitable to be classified by a decoding method in a single trial. The responses showed some characteristics of M190 responses and significantly varied among the body parts. Moreover, the significantly variable regions, which was shown with the high *F*-values of ANOVA, were located in the body-selective regions including EBA and FBA/FFA (Downing et al., 2001; Peelen and Downing, 2005; Thierry et al., 2006; Ishizu et al., 2010).

Using the MEG signals from 160 to 240 ms, the “categorical class” group was successfully classified with significant high accuracy compared with a chance level or “random class” group. This result suggests that the MEG signals from 160 to 240 ms selectively respond to categories of body parts and not only to a specific image of a body part.

The “categorical class” group includes category information of body parts, while the “random class” group does not. The presence of category information certainly contributed to the high accuracy of “categorical class.” As the accuracy of “categorical class” was significantly high from 160 to 240 ms, this suggests that the response at this time interval is involved in categorization of body parts. The timing of this response corresponds to the body-sensitive responses timing in previous studies (Thierry et al., 2006; Peelen and Downing, 2007; Ishizu et al., 2010; Meeren et al., 2013). Moreover, the responses of the EBA and FBA/FFA varied significantly among body-part categories with high *F*-values of ANOVA (**Figure 4**). These were consistent with the previous studies that have shown that the responses in these area were specific to the body parts (Bracci et al., 2010; Orlov et al., 2010). Overall, our study and previous studies suggest that body-sensitive responses at the EBA and FBA/FFA are involved in the discrimination of the body-part categories and the responses include category information of body parts.

Notably, among the three categories of body parts, the mouth category showed significant difference compared with the other categories. The variance of the cortical currents was evaluated between each pair of three categories: foot/mouth, hand/mouth, and foot/hand. The significant variance of cortical currents was observed around fusiform area with high *F*-values for the comparison of foot/mouth and hand/mouth. These results showed the responses to mouth around fusiform area were significantly different from those to foot and hand. These results are consistent with the previous studies demonstrating that the FFA responds selectively to human faces (Kanwisher et al., 1997; McCarthy et al., 1997; Gauthier et al., 1999; Engell and McCarthy, 2014). Thus, body sensitive responses occurring at the EBA and FBA/FFA possess enough information for classifying the body parts including face into the particular category.

Although previous studies in humans and non-human primates have shown body-selective neural responses in occipitotemporal areas (Kiani et al., 2007), our results demonstrate for the first time that the category information of the body parts can be non-invasively evaluated, with high temporal and spatial resolution, even in a single trial response. Using a decoding technique to analyze MEG signals, the representation and processing of visual information is determined at high spatiotemporal resolution. The differences in low-level properties, such as rough shapes for each body parts, might be responsible for decoding accuracies in this study. However, the high accuracy timing was close to 190 ms and responding regions for each body part were near to the EBA. Thus, it is the category information of body parts that was most likely to contribute to the decoding accuracy.

Notably, being able to decode categorical information, as in our study, is an important step in the context of brain–machine interface (BMI) research (Yanagisawa et al., 2011; Sugata et al., 2012). Successful decoding of a visual objective category demonstrates the possibility to infer the category of any arbitrary visual image. A decoder trained on a finite number of images cannot decode a random arbitrary visual image because there are infinite variations of images in the world. However, using our decoding method, we can infer the category of an image that was not used in the training of the decoder. Therefore, our method will contribute to enhance the BMI performance by providing arbitrary object recognition.

## Conflict of Interest Statement

The authors declare that the research was conducted in the absence of any commercial or financial relationships that could be construed as a potential conflict of interest.
